# The CORE (Consensus on Relevant Elements) Approach to Determining Initial Core Components of an Innovation

**DOI:** 10.3389/frhs.2021.752177

**Published:** 2021-11-16

**Authors:** Emily H. Kalver, D. Keith McInnes, Vera Yakovchenko, Justeen Hyde, Beth Ann Petrakis, Bo Kim

**Affiliations:** ^1^Department of Psychology, Montclair State University, Montclair, NJ, United States; ^2^Center for Healthcare Organization and Implementation Research, Veterans Health Administration, Bedford, MA, United States; ^3^Department of Health Law, Policy, and Management, Boston University School of Public Health, Boston, MA, United States; ^4^Section of General Internal Medicine, Boston University School of Medicine, Boston, MA, United States; ^5^Center for Healthcare Organization and Implementation Research, Veterans Health Administration, Boston, MA, United States; ^6^Department of Psychiatry, Harvard Medical School, Boston, MA, United States

**Keywords:** core components, consensus approach, intervention development, justice-involved veterans, community reintegration, behavioral health, social services, implementation evaluation

## Abstract

Identifying an intervention's core components is indispensable to gauging whether an intervention is implemented with fidelity and/or is modified; it is often a multi-stage process, starting with the first stage of identifying an initial set of core components that are gradually refined. This first stage of identifying initial core components has not been thoroughly examined. Without a clear set of steps to follow, interventions may vary in the rigor and thought applied to identifying their initial core components. We devised the CORE (Consensus on Relevant Elements) approach to synthesize opinions of intervention developers/implementers to identify an intervention's initial core components, particularly applicable to innovative interventions. We applied CORE to a peer-based intervention that aids military veterans with post-incarceration community reintegration. Our CORE application involved four intervention developers/implementers and two moderators to facilitate the seven CORE steps. Our CORE application had two iterations, moving through Steps 1 (individual core component suggestions) through 7 (group discussion for consensus), then repeating Steps 4 (consolidation of component definitions) through 7. This resulted in 18 consensus-reached initial core components of the peer-based intervention, down from the 60 that the developers/implementers individually suggested at Step 1. Removed components were deemed to not threaten the intervention's effectiveness even if absent. CORE contributes to filling a critical gap regarding identifying an intervention's initial core components (so that the identified components can be subsequently refined), by providing concrete steps for synthesizing the knowledge of an intervention's developers/implementers. Future research should examine CORE's utility across various interventions and implementation settings.

## Introduction

To successfully implement and spread interventions, it is essential to identify their core components for the purposes of fidelity, adaptation, replication, and evaluation. The U.S. Department of Health & Human Services defines core components as “essential functions or principles, and associated elements and intervention activities that are judged necessary to produce desired outcomes (1).” The notion of core components is key to implementation science, which focuses on promoting authentic adoption and replication of evidence-based interventions (2).

Such adoption requires ensuring that the intervention is implemented with fidelity—i.e., offering its identified core components (3). However, exact replication of the components across multiple implementation settings may be challenging (4) given the diverse and dynamic contexts that influence the feasibility of replication. This challenge is reflected in the growing focus on identifying and documenting adaptations (i.e., planned modifications) and unplanned modifications to interventions (5–9), so that resulting implementation and clinical outcomes can be understood in light of any deviations from the intervention's core components. Successfully identifying an intervention's core components is thus indispensable to gauging the extent to which an intervention is adopted and to assessing the modifications that were made for adoption. Identifying the core components of an intervention is not a simple task, however. Reviews of published literature on an intervention, when there is a sufficient body of articles, can shed some light on an intervention's core components (10–12). Without knowing an intervention's core components, it will be unclear during implementation, especially when it occurs across a range of contexts, which aspects of the intervention need to be maintained when making context-appropriate modifications (13, 14).

Identification of an intervention's core components is often a multi-stage process. First, the intervention developers, individuals with expertise regarding the implementation setting/context, implementers, or evaluators (or some combination) determine an initial (i.e., provisional) set of core components (henceforth, “initial core components”). These are then gradually refined as the intervention is implemented in multiple settings and contexts over time. Haynes et al. (15) offer a comprehensive test-and-refine process for identifying the core components of a new intervention. A critical element of the process' first stage is to inductively identify initial core components with input from both intervention designers and implementation evaluators. In this process, the evaluators draft the components then further develop them with the designers. How to conduct the process' first stage—i.e., how to identify initial core components—has not been thoroughly examined. Without a clear set of steps for this initial identification, different interventions may vary in the rigor and thought applied to identifying their initial core components. This may leave some interventions not well-specified, where, for instance, substantial differences may exist in what the intervention developers and implementers consider to be the core components.

**Figure 1:**
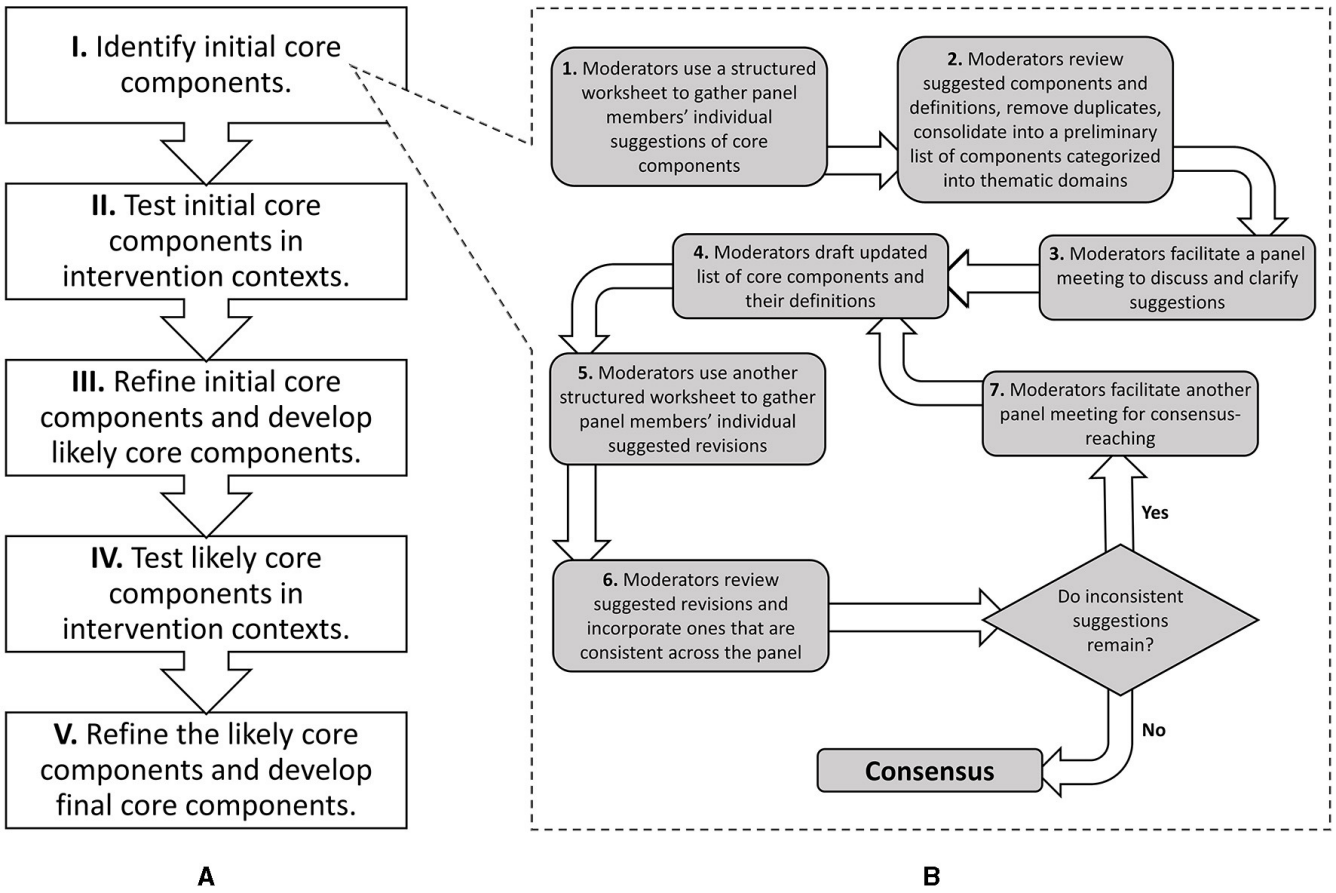
Depiction of where the CORE (Consensus on Relevant Elements) approach sits in the overall development and refinement of an intervention's core components. **(A)** Stages of the process of developing core components, adapted from Haynes et al. (15). **(B)** Flow of CORE steps for identifying initial core components.

To contribute to filling this gap, we devised a consensus group approach—the CORE (Consensus on Relevant Elements) approach—to gather and synthesize expert opinions to identify and refine an intervention's initial core components, particularly applicable to innovative interventions (henceforth, “innovations”) with limited empirical evidence. Specifically, guided by Landeta et al. (16)'s Hybrid Delphi methodology, innovation developers and implementers iteratively and systematically determine the initial core components. Figure 1 depicts where CORE sits in the overall development and refinement of an intervention's core components. We outline below each step of CORE then demonstrate CORE's application to specifying a peer support-based innovation that aids military veterans with community reintegration after their release from incarceration (17).

## Steps of the Consensus on Relevant Elements (CORE) Approach

Table 1 shows the CORE steps. CORE utilizes an expert panel and a moderator team. The expert panel consists of individuals knowledgeable about the purpose, context, and details of the innovation—typically the developers and implementers of the innovation. The moderator team typically consists of individuals experienced in facilitating group discussions toward consensus; their prior familiarity with the innovation can be helpful but is not required. The number of panel members and moderators should be large enough to (i) sufficiently represent expert knowledge about key aspects of the innovation and (ii) feasibly moderate the panel through the approach's seven iterative steps outlined in Table 1, respectively.

**Table 1:** Steps of the CORE (Consensus on Relevant Elements) approach to determining an innovation's initial core components.

Step 1	Moderators use a structured Worksheet A (Appendix 1 in Supplementary Material provides an example) to gather panel members' individual suggestions of core components into a table. Worksheet A prompts panel members to: •Suggest core components of the innovation •Define those components within the table
Step 2	Moderators review the suggested components and their definitions, remove duplicates, and consolidate them into a preliminary list of core components categorized into thematic domains—for example, categorized by the actions (e.g., training stakeholders on the innovation), entities (e.g., trainers, stakeholders), or timings (e.g., before/during/after training) indicated by the core components.
Step 3	Moderators facilitate a panel meeting to discuss and clarify overlapping/distinct suggestions of core components and their definitions.
Step 4	Moderators draft an updated list of core components and their definitions, based on the previous step's facilitated discussion. They structure this information into a Worksheet B (see Appendix 2 in Supplementary Material for an example), for panel members to individually complete as specified under Step 5.
Step 5	Each panel member independently fills out Worksheet B (created under Step 4) with their own suggested revisions to the updated list of core components and their definitions. Worksheet B prompts panel members to: •Revise the core components' definitions •Propose a short “code” (a brief phrase) for each component, to be used to refer to it in subsequent discussions •Suggest whether each component be split into multiple components, merged with another component, or moved under a different thematic domain •Suggest whether the thematic domain titles should be changed •Suggest whether each domain should be split into multiple domains or merged with another domain •Review questions tabled during the previous panel discussion, and suggest whether to address them as a part of determining the initial core components, or to revisit them following further innovation testing and implementation
Step 6	Moderators review the suggested revisions, incorporate ones that are consistent across the panel, and organize others into a list for further consideration (either as a part of determining the initial core components or to be revisited following further testing of the innovation's effectiveness and implementation).
Step 7	If inconsistent suggestions remain for considerations in determining the initial core components, moderators facilitate another panel meeting for consensus-reaching, then return to Step 4. If not, the latest list is considered to reflect the initial core components of the innovation.

## Context of the Post-Incarceration Engagement (Pie) Innovation

We applied CORE to determine the initial core components of the Department of Veterans Affairs (VA)'s Post-Incarceration Engagement (PIE) innovation (17). PIE uses peer specialists (“peers”) to enhance reentry support for veterans, extending the reach, duration, and intensity of support provided by the VA's Health Care for Reentry Veterans (HCRV) program. Peers are selected and hired for their “lived experience” that reflects many of the experiences of the reentry veterans, such as criminal justice involvement or recovery from mental illness and substance use disorders (SUDs). HCRV case managers work with veterans on an initial reentry plan and ensure they have housing and health care referrals upon release. There can be a warm handoff to the PIE peers (i.e., veteran is present when their HCRV case manager transfers their case to the PIE peer) who can work over a period of months with reentry veterans to enable their appointment attendance, both for VA health care (primary care, mental health, and/or SUD services) as well as to housing, employment, and other VA or community services as needed. To date, PIE has been implemented in two northeastern states in the United States, and is embarking on a larger implementation trial at six sites across four additional states through 2025. Hence, there was a pressing need to have consensus among the implementation team as to what the initial core components were, to ensure that at the subsequent sites there would be effective implementation, evaluation, and fidelity monitoring.

Section Application of CORE to Determine PIE's Initial Core Components outlines the detailed steps, and what was accomplished at each step, for our application of CORE to determine PIE's initial core components. The PIE implementation effort, of which this is a part, was submitted to the Institutional Review Board (IRB) at the VA Bedford Healthcare System (Massachusetts, USA), which determined it was a quality improvement project as per VA handbook 1200.05. The need for continued IRB review was waived.

## Application of “CORE” to Determine PIE's Initial CORE Components

Our application of CORE to determine PIE's initial core components involved a four-person expert panel and a two-person moderator team. Each expert panel member had extensive knowledge of PIE and its current evidence base, through developing, implementing, and/or evaluating the innovation. Both moderators were experienced facilitators of group discussions. One moderator did not have prior familiarity with PIE, while the other had continuously been a part of PIE implementation efforts. This CORE application had two iterations, first consecutively moving through Steps 1 through 7, then iterating back to repeat Steps 4 through 7. The steps are described below.

### Step 1

Using Worksheet A (Appendix 1 in Supplementary Material), the four panel members individually suggested, respectively, 11, 11, 17, and 21 core components (60 in total), along with their definitions, to be considered by the panel. To encourage original descriptions of the components, the worksheet deliberately did not require panel members to adhere to a rigid structure in describing their suggestions. There was considerable overlap among the four lists (see Step 2). Many of the definitions included wordings such as “if possible,” “ideally,” and “this often involves ….” For example, one suggestion was “If possible, the peer meets with the veteran on the day of the veteran's release from incarceration, to begin providing social and logistical support.” These wordings previewed that a main discussion point over the subsequent steps of the approach would be regarding whether a particular suggestion is a core component that is absolutely required for PIE or is a desirable but not essential feature.

### Step 2

The moderators reviewed the 60 suggestions gathered under Step 1, and removed clear duplicates. Then, they consolidated the remaining suggestions into a preliminary list of core components that grouped together thematically related or similar (but not entirely overlapping) suggestions. For example, three panel members suggested that training peers to support post-incarceration veterans is a core component of PIE, but there were differences in training content. These suggestions were grouped together in the consolidated list, with each proposed content presented side-by-side. After trying several different options for grouping the suggestions by actions (e.g., training stakeholders on the innovation), entities (e.g., trainers, stakeholders), or timings (e.g., before/during/after training), the moderators settled on grouping by the timings.

### Step 3

The moderators asked panel members to individually review the consolidated list from Step 2 before meeting as a panel. During the hour-long meeting, moderators facilitated discussions to identify individual aspects of each suggestion that are both essential to PIE (i.e., would fundamentally alter the nature of PIE if they could not be accomplished) and considered to be feasible for those practicing PIE. As an example, through discussions there was agreement that a core component was that the peer meet the veteran within the first 48 hours of release, without the strict requirement that the first meeting must occur on the day of release. One of the topics that came up most frequently was training of peers, for which four core components were proposed: training on PIE (what it involves and its underlying principles); VA health care system-required trainings; training for using the electronic health record (EHR) system (so peers can document in the EHR their encounters with veterans); and trainings to orient peers to correctional facilities' safety, security, and operations protocols (a requirement of many correctional facilities).

### Step 4 (First Iteration)

Based on Step 3, the moderators drafted an updated list of core components and their definitions. This updated list contained 20 core components grouped by three domains:

- (Domain I) Onboarding (hiring, orientation, and training) of peers, including
  ° What qualifications are looked for in a peer (e.g., experience with relevant VA services)
  ° What supervision of the peer involves (e.g., problem-solving challenges the peer faces)

- (Domain II) Peers' veteran-facing work, including
  ° Tasks before a veteran's release from incarceration (e.g., planning to meet post-release)
  ° Tasks after the release (e.g., linking the veteran to health care and other resources)

- (Domain III) Ongoing peer supervision, coordination, and networking, including
  ° Continued documentation in the EHR of the peer's encounters with the veteran
  ° Continued networking between the peer and services/resources relevant to the veteran

### Step 5 (First Iteration)

Using Worksheet B (Appendix 2 in Supplementary Material), the panel members individually suggested revisions to the list generated in the previous step. Suggested revisions included combining peer training on PIE with the training on the EHR system, and also combining the health care system-required/recommended trainings with trainings needed to access correctional facilities. An additional core component was suggested, regarding how the peer is expected to flexibly tailor their veteran-facing tasks based on the unique needs of the veteran.

### Step 6 (First Iteration)

Based on the previous step, the moderators updated the list of core components and their definitions. Changes included (i) emphasizing the need for the peer to remain up-to-date on both VA-based and non-VA-based services and resources relevant to the veteran, and (ii) checking that the core components related to ongoing tasks (e.g., continued supervision of the peer, from Domain III) are not redundant with the initiation of those tasks mentioned as core components under Domains I or II (e.g., establishing supervision procedures).

### Step 7 (First Iteration)

The moderators facilitated a second hour-long expert panel meeting to discuss additional suggestions of refining, combining, and/or de-duplicating core components. The panel members added the peer's tailoring of veteran-facing tasks to each veteran, and they discussed potential definitions for the component. They also moved the ongoing task of coordinating with the VA's existing justice outreach programs (in Domain III) to Domain I instead, where expectations for coordination are set as a part of onboarding. Component definitions and decisions of which domain each component belonged to were still not finalized at this point, so we iterated back to Step 4.

### Step 4 (Second Iteration)

Based on the previous step, the moderators drafted an updated list of core components and their definitions. The moderators added to Domain II the peer's tailoring of veteran-facing tasks to each veteran, with a working definition to be reviewed by the panel in the next step. The moderators clarified within the updated list that “supervision” refers to clinical supervision (separate from, for instance, guidance on the innovation that is provided to peers by individuals implementing PIE). Relatedly, the updated list emphasized that the clinical supervisor should be closely involved in decisions regarding how frequently the peer is to interact with the veteran.

### Step 5 (Second Iteration)

Using an updated Worksheet B reflecting the changes made in the previous step, the panel members individually suggested revisions to the updated list. These included specifying the peer's expected caseload of veterans, while keeping in mind the varied needs of veterans and expected differences across (i) geographic areas (e.g., an appropriate caseload for a peer may vary based on factors such as driving distances to correctional facilities and to veterans' housing) and (ii) peers' professional expertise (e.g., more experienced peers may be able to handle more cases simultaneously).

### Step 6 (Second Iteration)

Based on the previous step, the moderators updated the list of core components and their definitions. Changes included addressing the peer's expected caseload under Domain I, noting the peer's caseload from the first two states in which PIE was implemented. The previous step did not result in additional thoughts on whether core components related to ongoing tasks (Domain III) are redundant with the initiation of those tasks mentioned under Domains I or II (a topic of discussion under the first iteration's Steps 6 and 7, as noted above), so no changes were made yet to moving additional components away from Domain III.

### Step 7 (Second Iteration)

The moderators facilitated a third hour-long expert panel meeting to review the revised core components and their definitions. Two major decisions were made during this meeting. First, returning to the original conceptualization of training, the panel decided that there should be four distinct peer training-related core components, by decoupling peer training on PIE from the training on the EHR system, and also decoupling the health care system trainings from trainings needed to access correctional facilities (Appendix 3 in Supplementary Material shows the changes in the definitions of the training-related core components through our application of CORE, as an example of how the approach refines core components and their definitions). Second, Domain III was dissolved, following consensus among the expert panel that a separate domain was not necessary to represent the continuation of core components initiated under Domains I or II. Applying CORE resulted in the panel members agreeing that PIE consisted of 18 initial core components under two domains, as shown in Appendix 4 in Supplementary Material.

## Discussion

In this paper, we introduce CORE, an iterative consensus group approach relying on an expert panel and experienced moderators, to determine the initial core components of an innovation. The approach contributes to filling an existing gap in the literature on how to identify an innovation's initial core components, by providing a concrete sequence of steps for synthesizing the knowledge of an innovation's developers and implementers.

Our application of the approach has led to determining and specifying the initial core components of a VA innovation, Post-Incarceration Engagement (PIE), to assist veterans with community integration after incarceration. PIE is being spread to other sites around the United States, providing an important opportunity for the implementation team to record and analyze modifications that are made to adapt to local contexts (18). For innovations such as PIE that are in the midst of expanding their empirical evidence base, careful examination is warranted regarding (i) which of the initial core components need to be maintained as the innovation is modified to meet local needs, (ii) which modifications are enhancements to the core components, and (iii) which modifications are less desirable deviations from the core components, which may represent “program drift” (becoming a wholly different innovation) or “voltage drop” (a weakening of the active ingredients that make the innovation effective) (18).

Frameworks help assess modifications to core components – for instance, Wiltsey-Stirman et al.'s Framework for Reporting Adaptations and Modifications-Enhanced (FRAME) (5). Notably, CORE can be adapted for methodically incorporating expert opinions into assessing modifications. For example, per FRAME, determining the extent to which a modification is consistent with the core components, and/or the reason(s) for the modification, can be pursued through steps analogous to those that are described above. Namely, a moderator team can facilitate an expert panel to iteratively brainstorm, discuss, and reach consensus on both the nature of modifications to the innovation and whether the modifications suggest that core components need updating. Such discussion can also be useful for proactively planning future modifications prior to further implementation.

A potential limitation of CORE is that its utility could depend heavily on the moderators' meeting facilitation skills, and possibly also on their knowledge of the innovation and implementation science. This may increase the number of iterations through the steps, making the approach more time consuming, especially when panel consensus is difficult to reach. Additionally, as a novel approach, CORE has not been tested across various innovations and settings. However, given that the CORE steps are reliant neither on population nor content specifics of PIE, we expect that CORE can be applied to other health care innovations. CORE has also not been directly compared to other approaches that identify an innovation's initial core components. Thus, further work is needed to make such comparisons, and to apply CORE to a variety of innovations and in different health care settings. Accordingly, future enhancements to CORE may include (i) strengthening the validity of the expert panel's consensus through making explicit the panel's consideration of theories and mechanisms that link core components to desired outcomes and (ii) reflecting the field's evolving understanding of the extent to which core components may undergo context-specific modifications.

As rigorous methods are increasingly being applied in health care with guidance from implementation science, it is an opportune time to promote using a systematic approach for identifying core components that deliberately documents decisions made and makes explicit which components of an innovation are core, and which are desirable but would not threaten the innovation's effectiveness if they were absent. The CORE approach provides a systematic roadmap that innovation developers and implementers can follow to determine the initial core components, which can subsequently be tested and refined.

## Data Availability Statement

The original contributions presented in the study are included in the article/Supplementary Material, and further inquiries can be directed to the corresponding author.

## Ethics Statement

Ethical review and approval was not required for the study on human participants in accordance with the local legislation and institutional requirements. Written informed consent for participation was not required for this study in accordance with the national legislation and the institutional requirements.

## Author Contributions

EHK and BK led the writing of the manuscript and served as the expert panel moderators. DKM and BK initially conceptualized the project. VY provided consultation to the study team on expert panel procedures. DKM, VY, JH, and BAP served as the expert panel members and provided critical revisions to the manuscript's intellectual content. All authors contributed to the article and approved the submitted version.

## Funding

This work was funded by the U.S. Department of Veterans Affairs (VA) Health Services Research and Development Quality Enhancement Research Initiative (QUE 15-284).

## Author Disclaimer

The views expressed in this article are those of the authors and do not necessarily reflect the position or policy of the Department of Veterans Affairs or the United States government.

## Conflict of Interest

The authors declare that the research was conducted in the absence of any commercial or financial relationships that could be construed as a potential conflict of interest.

## Publisher's Note

All claims expressed in this article are solely those of the authors and do not necessarily represent those of their affiliated organizations, or those of the publisher, the editors and the reviewers. Any product that may be evaluated in this article, or claim that may be made by its manufacturer, is not guaranteed or endorsed by the publisher.
