# Supplementary material for: The CORE (Consensus on Relevant Elements) Approach to Determining Initial Core Components of an Innovation
Source: Front Health Serv. 2021 Nov 16;1:752177. doi: 10.3389/frhs.2021.752177 (PMC10012682; doi:10.3389/frhs.2021.752177)
Supplement: Supplementary file 1 [file Data_Sheet_1.docx]

Supplementary Material

# Appendix 1

**WORKSHEET A**

**Expert panel members’ individually brainstormed descriptions of the**

**Post-Incarceration Engagement (PIE) innovation’s core components**

*To be completed by expert panel members prior to the facilitated discussion on what the core components of the PIE innovation are*

Information requested of discussion participants:

- Please fill in the table below on what you consider to be core components of PIE.
- In filling out the table, please feel free to use your own wording to designate/describe/define the components or wording used in PIE’s source materials (e.g., protocol paper, innovation guide, etc.).
- Please add rows to the table as needed.

| **Core component** | **Definition (who, what, where, when, why)** |
| --- | --- |
|  |  |
|  |  |
|  |  |
|  |  |
|  |  |
|  |  |
|  |  |

*Thank you!*

# Appendix 2

**WORKSHEET B**

Expert panel members’ additional revision suggestions for the recently discussed and updated

core components of the Post-Incarceration Engagement (PIE) innovation

Background:

- Below are descriptions of 20 potential core components of PIE, preliminarily grouped into three domains.
- These descriptions are based on the recent facilitated expert panel discussion, supported by wordings within the pre-discussion document (Worksheet A) completed by each panel member.
- Please excuse any inaccurately captured descriptions. If you see any in this document, we ask that you please suggest corrections in your answers below. Highlighted items seek your additional input.

Instructions (Part 1 of 2):

- **For each of the 20 descriptions below, please briefly indicate:**

1. **How the description can be better worded**
2. **Whether the component should be further split into multiple components or merged with other components**
3. **What the component (or components, if suggesting a split into multiple components) being described should be called (i.e., a short “code” that can be used to refer to the component)**
4. **Whether the component(s) should be moved to be under a different domain**

|  | **How can this description be better worded?** | **Should this component be further split into multiple components? Merged with other components?** | **What should this component be called (give it a short “code” that we can use to refer to it)** | **Should this component be moved to be under a different domain? Which one?** |
| --- | --- | --- | --- | --- |
| Domain I: Onboarding (hiring, orientation, and training) of peers |  |  |  |  |
| 1. Peers are hired for PIE work, who are Veterans with (i) lived experience of incarceration and (ii) experience with a broad range of VA’s support programs (e.g., PLEASE PROVIDE EXAMPLES HERE). |  |  |  |  |
| 2. A clinical/implementation supervisor is identified for the peers, who will provide (at least weekly) supervision to support peers in (i) problem-solving challenges faced in PIE work, (ii) pursuing professional development, and (iii) addressing personal issues that the PIE work may bring up for them. |  |  |  |  |
| 3. Peers are trained in the contents of the PIE innovation manual, which include (i) elements of forensic reentry work, (ii) Whole Health concepts, (iii) outcomes of interest, and (iv) worksheets to be filled out with Veterans they support through PIE work (e.g., regarding goal setting and action planning). |  |  |  |  |
| 4. Peers complete required VA trainings through TMS, including for suicide prevention and using VA vehicles for transportation needs of their PIE work. Peers also undergo any additional standardized training activities for VA peer-support specialists (e.g., building skills in sharing one’s own story and confidentiality considerations). |  |  |  |  |
| 5. Peers are trained on entering data into CPRS, including learning the appropriate codes to apply when entering data on Veterans based on their PIE work. |  |  |  |  |
| 6. Peers complete requirements for accessing correctional facilities as a part of their PIE work (e.g., state may require peers to complete specific educational courses prior to granting access to the facilities). |  |  |  |  |

|  | **How can this description be better worded?** | **Should this component be further split into multiple components? Merged with other components?** | **What should this component be called (give it a short “code” that we can use to refer to it)** | **Should this component be moved to be under a different domain? Which one?** |
| --- | --- | --- | --- | --- |
| Domain II: Peers’ Veteran-facing work |  |  |  |  |
| 7.   Pre-release: The peer conducts preparatory activities prior to a Veteran’s release from incarceration. These may include (i) accompanying an HCRV specialist to meet with the Veteran, (ii) holding follow-up meetings/communication with the Veteran for planning to meet him/her on the day of release (or at least within 48 hours of release) to start providing social and logistical support, (iii) assisting with documents that the Veteran needs to get ready (e.g., for confirming eligibility and arranging housing), (iv) corresponding with legal, VA, and other entities (e.g., health care clinics, housing options) to coordinate appointments and additional preparations needed prior to release, and (v) getting a reentry kit of appropriate clothing and toiletries ready for the Veteran. |  |  |  |  |
| 8.   Day of and first 48 hours:  The peer meets with the Veteran on the day of release (or at least within 48 hours of release) to start providing social and logistical support, or as soon as possible if the Veteran joins the HCRV program only after being released from incarceration. |  |  |  |  |
| 9.   Early re-integration:  The peer checks in with the Veteran within the first two to four weeks of release (more or less frequently based on the Veteran’s needs/preferences SHOULD THIS CONCEPT OF CALIBRATING DOSE BE HERE OR IN A SEPARATE ROW) to keep the Veteran feeling supported during reentry and to be aware of how the Veteran is handling the change from incarceration. |  |  |  |  |
| 10.  Reintegration:  The peer conducts reentry planning with the Veteran based on Whole Health concepts. This includes discussing the Veteran’s goals and planning the steps needed to reach them, which take place at the start of the PIE work period and are updated after three months and after six months. Accordingly, the peer and the Veteran together complete the relevant worksheets in the PIE innovation manual. |  |  |  |  |
| 11.  Reintegration:  The peer helps the Veteran acquire basic skills needed for daily activities, such as using mobile technologies, using an ATM, figuring out transportation options, and planning meals. The peer also helps the Veteran build skills in communication skills for contacting legal, VA, and other entities (e.g., housing authorities, health care providers) as needed, including making a list of questions to ask and taking notes to document conversations. |  |  |  |  |
| 12.   Reintegration:  The peer links the Veteran to health care and other community resources, and, as needed, accompanies the Veteran to appointments for accessing these resources to help advocate for the Veteran. Especially important are linkages to services for physical/mental health, addiction recovery, and medication needs. |  |  |  |  |
| 13.   Reintegration:  The peer helps foster connections and build social support for the Veteran to other Veterans (e.g., by attending regular group meetings of reentry Veterans together with the Veteran) and to the HCRV program (e.g., by coordinating three-way meetings between the Veteran, the peer, and an HCRV specialist). |  |  |  |  |
| 14.  Sustaining momentum:  The peer maintains ongoing contact with the Veteran (GUIDELINES FOR HOW OFTEN?) for the duration of the PIE work, to provide continued social support, linkages, and role modeling. This may include continuing to encourage a healthy life style through helping to determine shared interest groups and other resources (e.g., fitness programs, religious institutions) that the Veteran can become a part of, and/or assisting with corresponding with family and friends if/when appropriate. |  |  |  |  |
| 15. Moving forward: The peer engages the Veteran in planning for the transition out of the PIE work period, as the Veteran gets established in his/her community and official reentry peer support through the PIE work nears its end. This includes reviewing the progress made through the PIE work period, identifying outstanding goals, and helping to link the Veteran to additional supportive resources (e.g., other VA peer support programs), ideally through a warm hand-off. Importantly, this also includes letting other professionals working with the Veteran know about the Veteran’s strengths and potential ongoing needs. |  |  |  |  |

|  | **How can this description be better worded?** | **Should this component be further split into multiple components? Merged with other components?** | **What should this component be called (give it a short “code” that we can use to refer to it)** | **Should this component be moved to be under a different domain? Which one?** |
| --- | --- | --- | --- | --- |
| Domain III: Ongoing peer supervision, coordination, and networking |  |  |  |  |
| 16.   Peers document what occurred during their encounters with Veterans that they are supporting through their PIE work. Peers enter relevant information into CPRS. |  |  |  |  |
| 17.   Peers meet weekly with their supervisor, during which progress on ongoing PIE work (including documented encounters) is reviewed, necessary adjustments are discussed, and upcoming tasks are planned. These meetings also regularly involve discussion of peers’ larger professional goals and steps needed to move towards achieving them. |  |  |  |  |
| 18.   Especially if the supervisor is not an HCRV specialist, peers regularly exchange information and network with members of the local HCRV program. SHOULD WE CONSOLIDATE #18-#20 INTO ONE NETWORKING ITEM WITH HCRV PROGRAM AND OTHER VA/NON-VA ENTITIES AS EXAMPLES? |  |  |  |  |
| 19.   Peers actively build and maintain relationships with legal entities and probation/parole officers with whom they need to collaborate for their PIE work. |  |  |  |  |
| 20.   Peers network with, introduce PIE to, and learn about allied efforts from, providers of reentry support outside VA, correctional facility leaders, and state departments offering services to Veterans. |  |  |  |  |

Instructions (Part 2 of 2):

- **For each of the three domains below, please indicate:**

1. **Whether the domain should be further split into multiple domains or merged with other domains**
2. **How the name of the domain (or domains, if suggesting a split into multiple domains) can be improved**

|  | Should this domain be split? Merged with other domains? | How can the name of the domain (or domains, if suggesting a split) be improved? |
| --- | --- | --- |
| Domain I: Onboarding (hiring, orientation, and training) of peers |  |  |
| Domain II: Peers’ Veteran-facing work |  |  |
| Domain III: Ongoing peer supervision, coordination, and networking |  |  |

# Appendix 3

**Change in the training-related core components’ definitions through the CORE (Consensus on Relevant Elements) approach, from pre- to post-consensus by the expert panel.** (CPRS: Computerized Patient Record System; DOC: Department of Correction; HCRV: Health Care for Reentry Veterans; HUD-VASH: U.S. Department of Housing and Urban Development-VA Supportive Housing; PIE: Post-Incarceration Engagement; PSS: Peer Support Specialist; TMS: Talent Management System; VA: Department of Veterans Affairs)

| *Pre-consensus suggestions for training-related core components, as phrased by each expert panel member* |
| --- |
| Train peer(s): Peers must be trained in VA systems as well as forensic reentry work. Training is ongoing throughout the intervention. [At the implementation site], peers used the intervention manual for training which included sections on evaluation and outcomes, Whole Health concepts and PIE worksheets. We also had discussions of ethics (confidentiality and peer support services) and stigma. They also watched training videos including video “A second chance for Veterans” https://www.youtube.com/watch?v=qJyuUq1AVVo and suicide prevention materials. Watched some VA peer training videos on communication, peer relationships, sharing your story, confidentiality, etc. Did required VA trainings including transportation so they can use VA car. Did CPRS training – both from VA and with [implementation team member] and got training from [clinical supervisor] on how to write notes appropriately. Took weeklong DOC course to gain access to prisons.  Peer trainings: PSS, forensic, TMS, etc.  Training on intervention model: Strategy includes reviewing PIE Guidance, reviewing sections and discussing over a multiple days, role playing, weekly or bi-weekly discussion of implementation issues, on-going monitoring and problem solving. Peers also need training on entering data in CPRS. This includes learning about the right codes to apply to the work so that Peer work is captured and useful to others. Recommend training on Suicide Prevention, VA policies on transportation, and completion of all other required VA trainings.  Training required to work in correctional facilities: Peers may be required to participate in trainings at correctional facilities so that they are approved to enter and meet with Veterans. |

| *Consensus-reached training-related core components, phrased as decided together by all expert panel members* |
| --- |
| Training – the PIE model and recovery work: Within the first month of hiring, peers (and HCRV specialists) commence training in the contents of the PIE intervention manual, which include (i) elements of forensic reentry work, (ii) Whole Health concepts, (iii) outcomes of interest, and (iv) conversational aids or tools to be filled out with Veterans they support through PIE work (e.g., regarding exploring Mission, Aspiration and Purpose, goal setting, and action planning; role-playing the use of these aids/tools could be helpful). Training will include discussions about how to tailor the core components of the PIE intervention to the local context. Training also covers reentry-related and VA-specific knowledge – e.g., the ins and outs of how state parole and probation work, the rules around what sex offenders must and must not do, learning and understanding the eligibility for VA programs (e.g., HUD-VASH, Domiciliary, detox, emergency beds, compensated work therapy), and being able to describe the programs to Veterans in a non-intimidating manner.  Training – VA-required and VA-recommended: Peers complete required and recommended VA trainings, including for suicide prevention and using VA vehicles for transportation needs for their PIE work. Peers also undergo any additional standardized training activities for VA peer-support specialists (e.g., building skills in sharing one’s own story and confidentiality considerations).  Training – documentation in the electronic health record: Peers are trained on the use the VA electronic health record system, including basic functions, appropriate codes for documentation of peer-related activities (e.g., entering brief encounter notes), timeframes for documentation, and use as a communication tool with Veterans’ healthcare team(s).  Training – requirements for accessing correctional facilities: Peers complete requirements for accessing correctional facilities as a part of their PIE work (e.g., state may require peers to take part in a facility orientation). |

# Appendix 4

Consensus of expert panel members on the core components of the

Post-Incarceration Engagement (PIE) innovation

*Latest update: 29 September 2020*

Background:

- Below are the current descriptions the core components of PIE.
- These descriptions have been drafted based on facilitated expert panel discussions and additional iterative feedback from the panel members.

Notes:

- This list of core components is not an ordered list of instructions.
- These components are applicable depending on the time at which the peer connects with the Veteran and the specific needs of the Veteran.

| **Domain I: Onboarding (hiring, orientation, and training) of peers** | |
| --- | --- |
| **1. Hiring PIE peer support specialists** | Peers are hired for PIE work, who are Veterans with (i) experience of recovery from social, emotional, and/or alcohol/substance use challenges and (ii) experience of using a broad range of VA’s support programs (e.g., mental health and substance use services, housing assistance, Compensated Work Therapy, benefits) to help improve/change their situation in a positive way. Hired peers must have a driver’s license and be eligible to enter correctional facilities. Ideally, peer support specialists have some experience with the criminal justice system. |
| **2. Identifying a clinical supervisor** | A clinical supervisor is identified for the peers, who is clinically licensed (e.g., social worker, psychologist) and will provide (at least weekly) supervision to support peers in (i) problem-solving challenges faced in PIE work through case reviews, (ii) pursuing professional development, and (iii) addressing personal issues that the PIE work may bring up for them. The supervisor is oriented to the PIE innovation, including review of the innovation guide to understand expected roles and responsibilities of PIE peers. Ideally, the supervisor would be familiar with issues related to Veterans with criminal-justice involvement. |
| **3. Training – the PIE model and recovery work** | Within the first month of hiring, peers (and HCRV specialists) commence training in the contents of the PIE innovation manual, which include (i) elements of forensic reentry work, (ii) Whole Health concepts, (iii) outcomes of interest, and (iv) conversational aids or tools to be filled out with Veterans they support through PIE work (e.g., regarding exploring Mission, Aspiration and Purpose, goal setting, and action planning; role-playing the use of these aids/tools could be helpful). Training will include discussions about how to tailor the core components of the PIE innovation to the local context. Training also covers reentry-related and VA-specific knowledge – e.g., the ins and outs of how state parole and probation work, the rules around what sex offenders must and must not do, learning and understanding the eligibility for VA programs (e.g., HUD-VASH, Domiciliary, detox, emergency beds, compensated work therapy), and being able to describe the programs to Veterans in a non-intimidating manner. |
| **4. Training – VA-required and VA-recommended** | Peers complete required and recommended VA trainings, including for suicide prevention and using VA vehicles for transportation needs for their PIE work. Peers also undergo any additional standardized training activities for VA peer-support specialists (e.g., building skills in sharing one’s own story and confidentiality considerations). |
| **5. Training – documentation in the electronic health record** | Peers are trained on the use the VA electronic health record system, including basic functions, appropriate codes for documentation of peer-related activities (e.g., entering brief encounter notes), timeframes for documentation, and use as a communication tool with Veterans’ healthcare team(s). |
| **6. Training – requirements for accessing correctional facilities** | Peers complete requirements for accessing correctional facilities as a part of their PIE work (e.g., state may require peers to take part in a facility orientation). |
| **7. Establishing expectations for communication with HCRV specialist** | Develop and implement expectations and plans for communication between the peer and the HCRV outreach specialist. This is especially important if the peer’s clinical supervisor is not the HCRV outreach specialist. The plan should include at least weekly communication about Veteran cases and time to share information and problem solve. |
| **8. Determining peers’ target caseload** | Determine the peers’ target active caseload, with input from the HCRV specialist and the clinical supervisor, recognizing that (i) Veterans will vary in their needed amount of peer contact and (ii) a feasible caseload at one site may not be feasible at another site (e.g., when considering differences in numbers of Veterans released from incarceration, driving distances, etc.). In pilot work, peers’ caseloads were 10-15 cases at a time. |

| **Domain II: Peers’ Veteran-facing work** | |
| --- | --- |
| **9. Pre-release activities** | Pre-release: The peer works with the HCRV outreach specialist to conduct preparatory activities prior to a Veteran’s release from incarceration. These may include (i) communication with prison or jail reentry specialists to confirm day and time of release (ii) accompanying an HCRV specialist to meet with the Veteran, (iii) assisting with identification of housing options and plans during the initial period of reentry, (iv) assistance with applying for and securing documents that may be needed to carry out reentry plans (e.g., DD 214, birth certificate), (v) corresponding with legal, VA, and other entities (e.g., health care clinics, housing options) to coordinate appointments and additional preparations needed prior to release, (vi) assisting with plans for transportation on the day of release, (vii) holding follow-up meetings/communication with the Veteran to start providing social and logistical support, and (viii) getting a reentry kit of appropriate clothing and toiletries ready for the Veteran, and/or (ix) communicating with family members about the release. |
| **10. Day of release and first 48 hours** | Day of release and first 48 hours: The peer meets with the Veteran on the day of release (or at least within 48 hours of release) to start providing social and logistical support. Providing social and logistical support immediately upon release can help reduce anxiety, build trust, and prevent negative outcomes known for this vulnerable period. Transportation from the facility to their residence and other required check-ins (probation, parole, local police station) is a critical need on the day of release. Peers can also assure that basic needs are met during this initial period, including linkage to clothing, food, hygiene resources. Providing logistical support (prioritizing getting an identification / social security card; signing up for health insurance, getting a VA primary care provider, etc.) should also happen as soon as possible, although it may happen after the first 48 hours. If a Veteran connects with the HCRV program after release, assessing and addressing these basic needs as quickly as possible is recommended. |
| **11. First few weeks of reentry** | During the first few weeks of reentry, the peer, with guidance from their clinical supervisor, focuses on developing a trusting relationship with the Veteran and calibrating Veteran’s needs. This includes providing social and logistical support to meet priority needs and engaging the Veteran in conversations that will help with future planning and support. Strategies for relationship building include the following:   - During the first two weeks of reentry, meetings may be more frequent to assure that the Veteran is getting established in their new residence and able to meet their basic needs. Ideally the peer has at least two in-person meetings per week during this period and communication via phone/text as needed. Meeting frequency will likely vary over time given the Veteran’s circumstances. - There is checklist of things the Veteran may need to get reestablished in the community in the PIE innovation guide. Within the first two weeks, discuss the items on the checklist, identify which ones are needed, and begin working to address those needs. |
| **12. Reentry planning for Whole Health** | The peer conducts reentry planning using a Whole Health approach to support the Veteran as they begin the process of social reintegration. The approach ideally begins with the Whole Health Personal Health Inventory, which is a reflective exercise about what matters most to Veterans. This reflection is a springboard for reentry planning which includes setting goals and establishing clear action steps that will help the Veteran prioritize their efforts. This reflection and planning process may take place over a series of encounters. These goals are not static, and progress should be reviewed regularly. |
| **13. Skill building and problem solving** | The peer helps the Veteran acquire basic skills needed for daily activities, such as using mobile technologies, using an ATM, figuring out transportation options, and planning meals. The peer also helps the Veteran build communication and self-advocacy skills for contacting legal, VA, and other entities (e.g., housing authorities, health care providers) as needed. The peer is a role model. This may include activities like making a list of questions to ask, taking notes to document conversations, being persistent when contacting an organization or agency for assistance (may require multiple phone calls, or visits get an ID, secure bank account, etc.), being on time to meetings, etc. |
| **14. Linkage to VA and community resources** | The peer links the Veteran to health care and services in the VA and in the community that help him/her progress towards personal goals and supports reintegration back into community settings. Especially important are linkages to services for physical/mental health, addiction recovery, and medication needs – the peer helps assure that the Veteran has initial physical and mental health appointments at VA or in the community. The peer helps the Veteran learn about and take advantage of what the community has to offer. This could also include social/recreational aspects of the community (e.g., religious institutions, sports clubs, parks, senior centers, etc.). As needed, the peer offers tangible support to connect with and access services and resources, such as transportation, appointment accompaniment, and assistance making appointments. |
| **15. Strengthening social networks and support** | The peer helps build social support for the Veteran and foster connections to other Veterans (e.g., by attending regular group meetings, introducing the Veteran to other Veterans, and encouraging community involvement; peers may also start a group for justice-involved Veterans if one does not exist and it is feasible to do so). |
| **16. Ongoing contact and assessment** | Ongoing contact and assessment:   - The peer maintains ongoing contact with the Veteran for approximately 4-6 months and provides social support, linkages, and role modeling. This may include continuing to encourage a healthy lifestyle through helping to determine shared interest groups and other resources (e.g., fitness programs, religious institutions) that the Veteran can become a part of, and/or assisting with corresponding with family and friends if/when appropriate. - The peer conducts on-going assessment of the Veteran’s interests and needs, thinking ahead about the kinds of resources and supports that he/she may need to help move towards independence. The peer may also ask the Veteran to review and update their Personal Health Inventory as part of the ongoing assessment process. If the Veteran is likely to need intensive support for longer than the typical 4-6 months that PIE is designed for, the peer discusses with the case manager and other healthcare team members about available resources. Transitions (see #17 below) often take time and may need planning ahead to allow for new relationships to be built. |
| **17. Transitional support** | The peer engages the Veteran in planning for the transition out of more intensive PIE peer support, as the Veteran gets established in his/her community. This includes reviewing the progress made through the PIE work period, identifying outstanding goals, and helping to link the Veteran to additional supportive resources (e.g., other VA peer support programs such as in HUD-VASH long-term supportive housing), ideally through a warm hand-off. This also includes letting other professionals working with the Veteran know about the Veteran’s strengths and potential ongoing needs, following discussion with the Veteran regarding what information is appropriate to share. |
| **18. Networking for collaboration** | Peers actively build and maintain relationships with VA, legal, and community providers with whom they need to collaborate to support Veterans. This may be through (i) attending local or regional meetings with collaborating partners, (ii) meeting with each collaborating partner to learn about their work, provide an overview of the PIE program, and discuss how they can collaborate to assist them (iii) joining reentry task forces, and/or (iv) volunteering at events sponsored by collaborating partners. Examples of relationships that may important to build and maintain include probation/parole officers, transitional housing programs, HUD-VASH case managers, and correctional facility reentry personnel. |
